# Ambroxol Hydrochloride Combined with Fluconazole Reverses the Resistance of *Candida albicans* to Fluconazole

**DOI:** 10.3389/fcimb.2017.00124

**Published:** 2017-04-07

**Authors:** Xiuyun Li, Yuanhao Zhao, Xin Huang, Cuixiang Yu, Yilei Yang, Shujuan Sun

**Affiliations:** ^1^School of Pharmaceutical Sciences, Shandong UniversityJinan, China; ^2^Pharmaceutical Department, Qianfoshan Hospital Affiliated to Shandong UniversityJinan, China; ^3^Respiration Medicine, Qianfoshan Hospital Affiliated to Shandong UniversityJinan, China

**Keywords:** ambroxol hydrochloride, fluconazole, drug resistance, *Candida albicans*, drug uptake and efflux

## Abstract

In this study, we found that ambroxol hydrochloride (128 μg/mL) exhibits synergistic antifungal effects in combination with fluconazole (2 μg/mL) against resistant planktonic *Candida albicans* (*C. albicans*) cells. This combination also exhibited synergistic effects against resistant *C. albicans* biofilms in different stages (4, 8, and 12 h) according to the microdilution method. *In vitro* data were further confirmed by the success of this combination in treating *Galleria mellonella* infected by resistant *C. albicans*. With respect to the synergistic mechanism, our result revealed that ambroxol hydrochloride has an effect on the drug transporters of resistant *C. albicans*, increasing the uptake and decreasing the efflux of rhodamine 6G, a fluorescent alternate of fluconazole. This is the first study to investigate the *in vitro* and *in vivo* antifungal effects, as well as the possible synergistic mechanism of ambroxol hydrochloride in combination with fluconazole against resistant *C. albicans*. The results show the potential role for this drug combination as a therapeutic alternative to treat resistant *C. albicans* and provide insights into the development of antifungal targets and new antifungal agents.

## Introduction

In the past few decades, fungi have emerged as an important cause of severe and fatal infections, with a >200% increase in annual fungal sepsis cases recorded in the USA (Pfaller and Diekema, 2007). *C. albicans* is one of the most common opportunistic pathogens in humans, and the infections caused by this fungus are associated with a high mortality, often in excess of 30%, due to the limited types of available antifungal agents and the emergence of drug resistance (Wisplinghoff et al., 2004; Pfaller and Diekema, 2007). Only a few types of antifungal agents are currently available, such as azoles, echinocandins, polyenes, and allylamines (Prasad et al., 2016). Among them, fluconazole (FLC), one of the azoles, is the most commonly used agent to treat infections caused by *C. albicans* due to its high bioavailability and low toxicity (Fiori and Van Dijck, 2012; Han et al., 2013; Sarkar et al., 2014). However, the extensive and frequent use of FLC has led to the development of genetically acquired drug resistance (e.g., overexpression of drug efflux genes) in *C. albicans* and has introduced challenges in the treatment of fungal infections. In addition, *C. albicans* can easily form biofilms on medical devices, which is considered physiologically associated with the acquisition of drug resistance and results in the resistance of *C. albicans* to antifungal agents (Mathe and Van Dijck, 2013; Zhang et al., 2013; Akbari and Kjellerup, 2015). Therefore, drug resistance in *C. albicans* is becoming an increasingly important issue. Treatment approaches for infections caused by drug-resistant *C. albicans* are lacking, and patients must receive pharmacotherapy with toxic antifungals (Chen et al., 2013). Thus, the development of improved antifungal treatments to combat drug resistance is urgently needed. Combination treatment of FLC with non-antifungal agents has been one of the most successful approaches to combat the resistance of fungi (Liu et al., 2014, 2016; Gu et al., 2016). Utilizing synergistic drug combinations could potentially reduce the toxicity that may occur when patients are exposed to high doses of drugs.

Ambroxol hydrochloride (ABH) is widely used to treat chronic obstructive pulmonary disease, chronic bronchitis, bronchial asthma, spastic bronchitis, and bronchopulmonary complications after chest surgery. ABH is associated with potent expectorating action, improvement of respiratory function and low toxicity (Malerba and Ragnoli, 2008). Furthermore, ABH has been reported to exert antioxidant activity and relieve inflammation by reducing the release of inflammatory cytokines (Gillissen et al., 1997; Pfeifer et al., 1997; Gibbs et al., 1999). The efficacy, tolerability and safety of ABH have been clinically verified (Fischer et al., 2002; Malerba and Ragnoli, 2008; de Mey et al., 2015). Interestingly, ABH alone at high concentrations from 1 to 60 mg/mL has been shown to exhibit antimicrobial effects on *C. albicans, Candida parapsilosis* and *Pseudomonas aeruginosa* (Li et al., 2008; Pulcrano et al., 2012; Rene et al., 2014). However, no study has investigated the combined antifungal effects of ABH with FLC.

The present study was designed to determine the potential antifungal effects of FLC and ABH against *Candida* spp. and the effects of this drug combination on resistant *C. albicans* biofilms in different stages *in vitro*. A recently established infection model, *Galleria mellonella* (*G. mellonella*), was used to determine the antifungal effects of this drug combination *in vivo*. The potential synergistic mechanism associated with this drug combination was also detected by determining the activity of drug uptake and efflux of resistant *C. albicans*.

## Materials and methods

### Isolates and growth media

Twelve *Candida* spp. isolates with different susceptibilities were used in this study, including four *C. albicans* isolates (CA4, CA8, CA10, and CA16), four *Candida glabrata* isolates (CG1, CG2, CG3, and CG8) and four *Candida krusei* isolates (CK2, CK3, CK9, and CK10). Both *C. glabrata* and *C. krusei* are non*-C. albicans* types. Each *Candida* spp. included two resistant and two susceptible isolates, as shown in Table 1. *Candida albicans* ATCC 10231 was used as a quality control isolate. All isolates were refreshed from the frozen storage at −80°C and inoculated twice onto yeast–peptone–dextrose (YPD) solid medium (2% agar, 2% peptone, 2% glucose, and 1% yeast extract) for 18 h at 35°C. YPD liquid medium consisted of 2% peptone, 2% glucose, and 1% yeast extract. Egg yolk agar medium consisted of 5% NaCl, 1% peptone, 3% glucose, 0.0006% CaCl_2_, 10% egg yolk, and 2% agar.

**Table 1:** **Drug interactions of FLC and ABH against ***Candida*** spp. ***in vitro*****.

**Drugs**	**Isolates^a^**	**MIC (**μ**g/mL)**^b^				**FICI model**	
		**FLC**	**FLC_comb_**	**ABH**	**ABH_comb_**	**FICI^c^**	**Interpretation**
	CA4 (S)	1	0.0313	>512	512	1.031	No interaction
	CA8 (S)	2	0.25	>512	512	1.125	No interaction
	CA10 (R)	>512	2	512	128	0.254	Synergism
	CA16 (R)	>512	2	512	128	0.254	Synergism
	CG1 (S)	16	0.25	512	512	1.016	No interaction
FLC+ABH	CG8 (S)	32	16	512	256	1.000	No interaction
	CG2 (R)	128	32	512	256	0.750	No interaction
	CG3 (R)	128	0.5	512	512	1.004	No interaction
	CK2 (S)	16	8	512	512	1.500	No interaction
	CK3 (S)	32	2	512	512	1.063	No interaction
	CK9 (R)	64	32	512	256	1.000	No interaction
	CK10 (R)	256	32	1024	512	0.625	No interaction

a *Twelve isolates with different susceptibilities involved three Candida spp. CA, Candida albicans; CG, Candida glabrata; CK, Candida krusei*.

b *MICs denote the MIC_80_ of each drug alone or in combination (comb) against the isolates and are shown as the median of three independent experiments; FLC, fluconazole; ABH, ambroxol hydrochloride; S, susceptible; R, resistant*.

c *The FICI value is the median of three independent experiments; FICI ≤ 0.5 for synergism, FICI > 4.0 for antagonism and 0.5 < FICI ≤ 4.0 for no interaction*.

### Drugs and *G. mellonella* larvae

All drugs, including FLC, ABH, and ampicillin, were purchased from Dalian Meilun Biotech Co., Ltd. (Dalian, China). Stock solutions of FLC (2,560 μg/mL), ABH (5,120 μg/mL), and ampicillin (2,560 μg/mL) were prepared in sterile distilled water and stored at −20°C until use. The larvae of *G. mellonella* were purchased from Tianjin Huiyude Biotech Co., Ltd. (Tianjin, China).

### Determination of *In vitro* antifungal effects

#### Determination of the minimal inhibitory concentration (MIC) of planktonic cells

The MICs of FLC and ABH against 12 *Candida* spp. isolates were determined with a broth microdilution method using two-fold serial dilutions in RPMI 1640 medium as described by the Clinical and Laboratory Standards Institute method M27-A3 document. The tests were performed in 96-well flat-bottomed microtiter plates. Plates were incubated at 35°C for 24 h. After incubation, an XTT reduction assay was used to determine the MIC, which was read at the lowest concentration that showed 80% inhibition of growth compared with that of the drug-free control (Li et al., 2015). The fractional inhibitory concentration index (FICI) model was used to interpret the antifungal effects on planktonic cells *In vitro*. The FICI model is as follows (Guo et al., 2008): FICI = FIC_FLC_ + FIC_ABH_ = (MIC_80_ of FLC in combination/MIC_80_ of FLC alone) + (MIC_80_ of ABH in combination/MIC_80_ of ABH alone). The results were defined as FICI ≤ 0.5 for synergism, FICI > 4.0 for antagonism and 0.5 < FICI ≤ 4.0 for no interaction (Odds, 2003).

#### Determination of sessile MIC (SMIC) of *C. albicans* biofilms

The SMICs of FLC and ABH against resistant *C. albicans* biofilms were tested as described previously (Ramage and Lopez-Ribot, 2005). As described in a previous study, 4, 8, and 12 h biofilms of CA10 were formed in 96-well flat-bottomed microtiter plates (Gu et al., 2016). Then, drugs were added to the plates and the plates were incubated for an additional 24 h at 37°C. XTT reduction assays were performed to determine the SMICs. SMICs were defined as the lowest antifungal concentrations where there was an 80% (SMIC_80_) reduction in the XTT-colorimetric readings in comparison to the drug-free control. Colorimetric changes were measured at 492 nm with a microtiter plate reader (SpectraMax190, USA). The FICI model was used to interpret the antifungal effects for biofilms as described above.

### Determination of *In vivo* antifungal effects

Batches of *G. mellonella* larvae in the final instar stage were stored in the dark at 25°C, and larvae weighing approximately 250 mg were selected for use in the experiments. The doses (1.6 μg/larva for FLC and 3.2 μg/larva for ABH) used to treat larvae mimicked those used to treat humans. Each group contained 16 randomly chosen larvae. The preliminary steps for each *in vivo* experiment were the same. In detail, larvae were inoculated with 10 μL of the cell suspension (5 × 10^8^ CFU/mL) into the prolegs using 50- μL syringes (Shanghai Gaoge Biotech Co., Ltd. Shanghai, China). After 2 h, treatment with the drugs separately or in combination was administered using the 50- μL syringes, while a control group of infected larvae received PBS only.

For survival assays, a preliminary experiment was first carried out to test whether there are harmful effects of PBS and drugs on the uninfected *G. mellonella* larvae. Only 10 μL of PBS, FLC, ABH, and drug combination were inoculated into the *G. mellonella* larvae in each group, without treatment of the CA10 cell suspension. For the formal survival assay, the larvae were pretreated as described above. The number of surviving larvae in each group was recorded every day for up to 4 days. Survival data were plotted according to the Kaplan-Meier method using the Statistical Product and Service Solutions 19 software.

For determination of the fungal burden, the larvae were pretreated as described above. Three larvae from each group were randomly culled every day over 4 days. *C. albicans* burdens were determined by inoculating dilutions of the homogenized larvae onto YPD solid medium plates as previously described (Gu et al., 2016). These plates were incubated overnight at 35°C. Fungal burden data were recorded using a CFU counting method.

For histological study, the larvae were pretreated as described above. One infected larva from each group was randomly selected at 3 days post-infection and cut into 7-μm tissue slices. The tissue slices of infected larvae were stained with Periodic acid Schiff reagent (PAS) and observed under an Olympus FSX100 fluorescence microscope with 4.2 × 10 objectives.

### Synergistic mechanism explorations

#### Uptake and efflux of rhodamine 6G (Rh6G)

CA10 cells at a concentration of 1 × 10^6^ CFU/mL were allowed to grow aerobically in 5 mL YPD liquid medium at 35°C for 18 h. The cells were harvested, washed twice with glucose-free PBS, and resuspended in the same buffer to obtain a concentration of 1 × 10^7^ CFU/mL. The cells were then de-energized for 1 h in glucose-free PBS. The de-energized cells were harvested, washed, and then resuspended again to obtain a concentration of 1 × 10^7^ CFU/mL.

To assess the uptake of Rh6G, Rh6G, and ABH were added to the exhausted cells at a final concentration of 10 μM and 128 μg/mL, respectively. Cells without ABH treatment served as the control. Samples were incubated for 50 min at 35°C and then washed with glucose-free PBS. A BD FACSAria II flow cytometer (Becton Dickinson, USA) with excitation at 488 nm and emission at 530 nm was used to determine the mean fluorescent intensity (MFI) of each sample at specific time intervals (0, 10, 20, 30, 40, and 50 min).

To assess the efflux of Rh6G, Rh6G was added to the exhausted cells at a final concentration of 10 μM. Cells were then washed with glucose-free PBS, and ABH was added to the cells at a final concentration of 128 μg/mL. Cells without ABH treatment served as the control. Samples were incubated for 50 min at 35°C and then washed with glucose-free PBS. After the addition of 5 mL of 5% glucose-PBS to each sample, the MFI was immediately determined using the flow cytometer as described above at specific time intervals (0, 30, 60, 90, 120, and 150 min).

## Results

### ABH combined with FLC exhibited synergistic effects on planktonic cells of resistant *C. albicans In vitro*

To assess the *in vitro* interactions between FLC and ABH against *Candida* spp., the data were obtained using the broth microdilution method and interpreted using the FICI model. The MICs of FLC and ABH alone and in combination against the 12 *Candida* spp. isolates *in vitro* are listed in Table 1. The results show that ABH combined with FLC has synergistic effects on resistant *C. albicans in vitro*. The addition of ABH resulted in a decrease in the MIC of FLC for resistant *C. albicans* CA10 and CA16 from >512 to 2 μg/mL, while both of the FICI values were lower than 0.5 (Table 1, Table S1). However, no synergism was observed when this drug combination was used to inhibit two susceptible *C. albicans* isolates and eight non-*C. albicans* isolates *in vitro*, with the FICIs distributed from 0.625 to 1.500 (Table 1).

### ABH combined with FLC exhibited synergistic effects against biofilms in different stages

To assess interactions between FLC and ABH against *C. albicans* biofilms at different stages, the data obtained by the broth microdilution method were analyzed using the FICI model described above. The results for the tested drugs alone and in combination in biofilms of resistant *C. albicans* at 4, 8, and 12 h are summarized in Table 2. There was close agreement between these two models for the FLC-ABH combination treatment on *C. albicans* biofilms. As shown in Table 2, ABH combined with FLC showed synergistic antifungal effects against 4, 8, and 12 h biofilms, all with FICI = 0.252. In addition, the MIC of ABH for 12 h biofilms (256 μg/mL) was higher than that of 4 and 8 h biofilms (128 μg/mL).

**Table 2:** **Synergistic effects of FLC alone and in combination with ABH against biofilms of resistant ***C. albicans*****.

**Time (h)^a^**	**SMIC(**μ**g/mL)**^b^				**FICI model**	
	**FLC**	**FLC_comb_**	**ABH**	**ABH_comb_**	**FICI^c^**	**Interpretation**
4	>1024	2	512	128	0.252	Synergism
8	>1024	2	1024	256	0.252	Synergism
12	>1024	2	1024	256	0.252	Synergism

a *Time indicates the incubation period of biofilm formation*.

b *SMICs denote the SMIC_80_ of each drug alone or in combination (comb) against the biofilms and are shown as the median of three independent experiments. FLC, fluconazole; ABH, ambroxol hydrochloride*.

c *The FICI value is the median of three independent experiments; FICI ≤ 0.5 for synergism, FICI > 4.0 for antagonism and 0.5 < FICI ≤ 4.0 for no interaction*.

### ABH combined with FLC showed synergistic effects against resistant *C. albicans in vivo*

**Figure 1:**
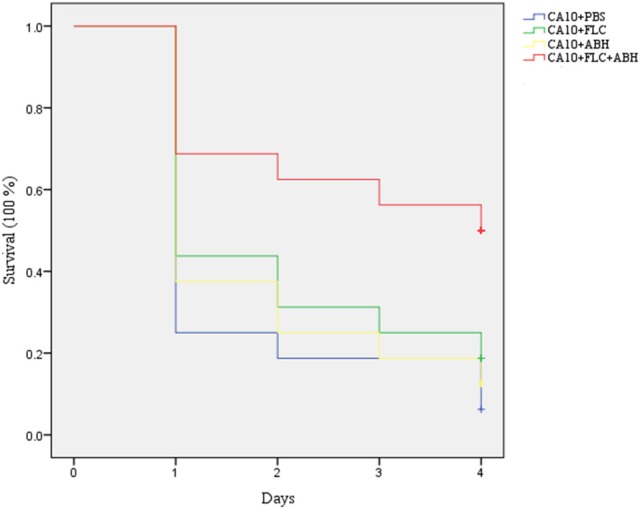
**Effect of FLC alone or in combination with ABH on the survival of infected ***G. mellonella*** over 4 days**. The concentration of yeast cells was 5 × 10^6^ CFU/larva. Treatments consisted of PBS, FLC (1.6 μg/larva) alone, ABH (3.2 μg/larva) alone, or a combination of FLC (1.6 μg/larva) with ABH (3.2 μg/larva). The Statistical Product and Service Solutions 20 software was used to analyze the data. The experiments were performed three times on different days.

**Figure 2:**
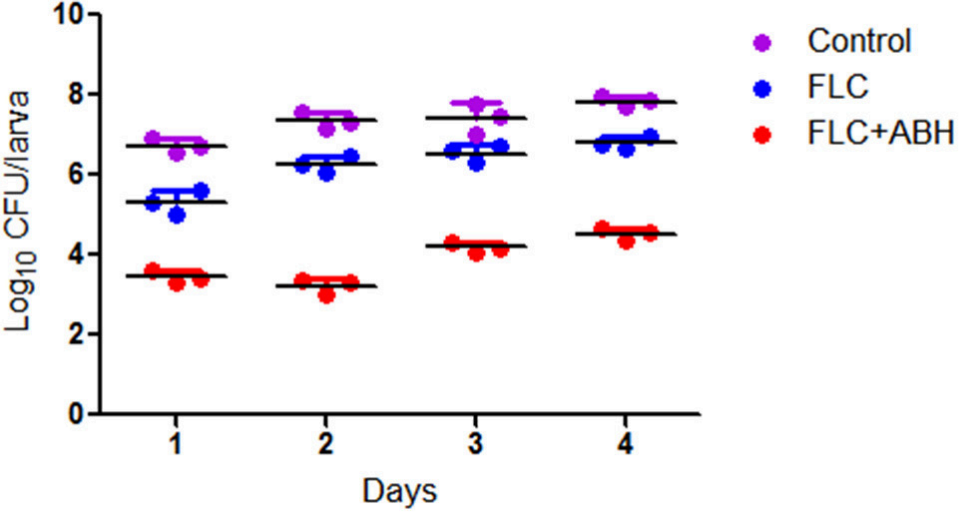
**Effect of FLC alone or in combination with ABH on the fungal burden of infected ***G. mellonella*** over 4 days**. The concentration of yeast cells was 5 × 10^6^ CFU/larva. Treatments consisted of PBS, FLC (1.6 μg/larva) alone, ABH (1.6 μg/larva) alone, or a combination of FLC (1.6 μg/larva) with ABH (1.6 μg/larva). For clarity, data for the treatment with ABH are not shown because the data closely followed those of the control group. GraphPad Prism 6 software was used to analyze the data. The experiments were performed three times on different days.

**Figure 3:**
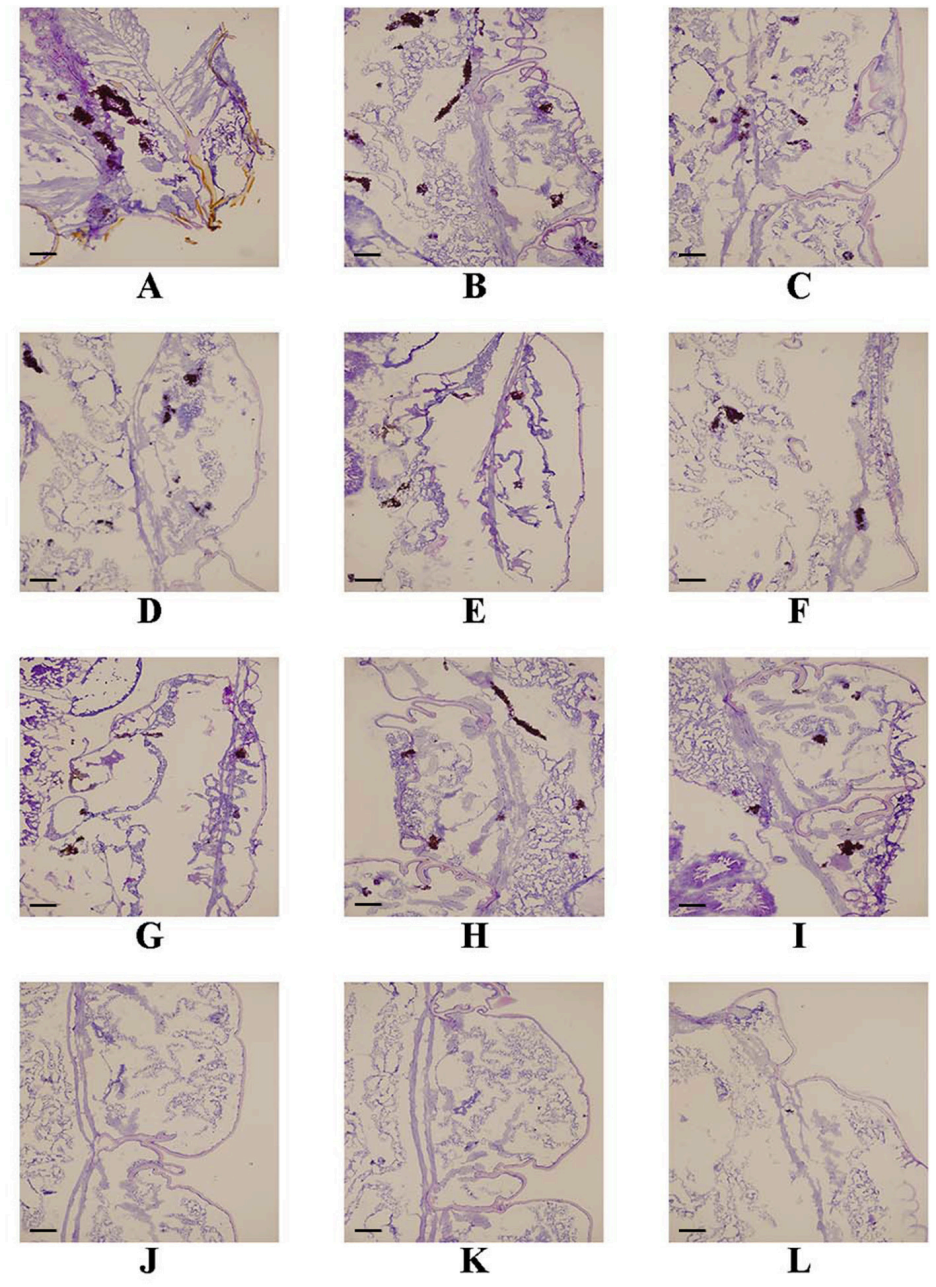
**Effect of FLC alone or in combination with ABH on the histopathology of infected ***G. mellonella*** at day 2 post-infection**. The concentration of yeast cells was 5 × 10^6^ CFU/larva. Treatments consisted of PBS **(A–C)**, FLC (1.6 μg/larva) alone **(D–F)**, ABH (1.6 μg/larva) alone **(G–I)**, or a combination **(J–L)** of FLC (1.6 μg/larva) with ABH (1.6 μg/larva). Melanized nodules consisted of mixtures of yeast cells and filaments. Bar = 200 μm. The experiments were performed three times on different days.

Following the identification of the synergism between FLC and ABH *in vitro*, experiments were designed to identify whether this effect would be replicated *in vivo*. In the preliminary survival assay, all uninfected *G. mellonella* larvae in four groups (PBS, FLC, ABH, and drug combination) were alive after 4 days, indicating that PBS and the drugs have no harmful effect on *G. mellonella*. The effect of the drugs alone or in combination on the survival rates, fungal burden and histopathology of infected larvae is shown in Figures 1–3, respectively. When used alone, the drugs, FLC and ABH had no significant effect on the survival rates of infected larvae. However, their combination significantly improved the survival rates, confirming the efficacy of this drug combination *in vivo* (Figure 1). Encouragingly, the combination of FLC and ABH resulted in a greater reduction in fungal burden compared to the control and individual drug groups, as shown by the slower proliferation of *C. albicans* in the group receiving the drug combination over 4 days (Figure 2). Slices of infected larvae in four groups revealed a significant decrease in the number of melanized nodules in larvae treated with this drug combination, compared to no drug or larvae treated with individual drugs (Figure 3). The histopathology results are in agreement with the survival rates and fungal burden experiments, which further confirms the synergistic effects of FLC and ABH combination against resistant *C. albicans in vivo*.

### ABH inhibited the activity of drug transporters of resistant *C. albicans*

**Figure 4:**
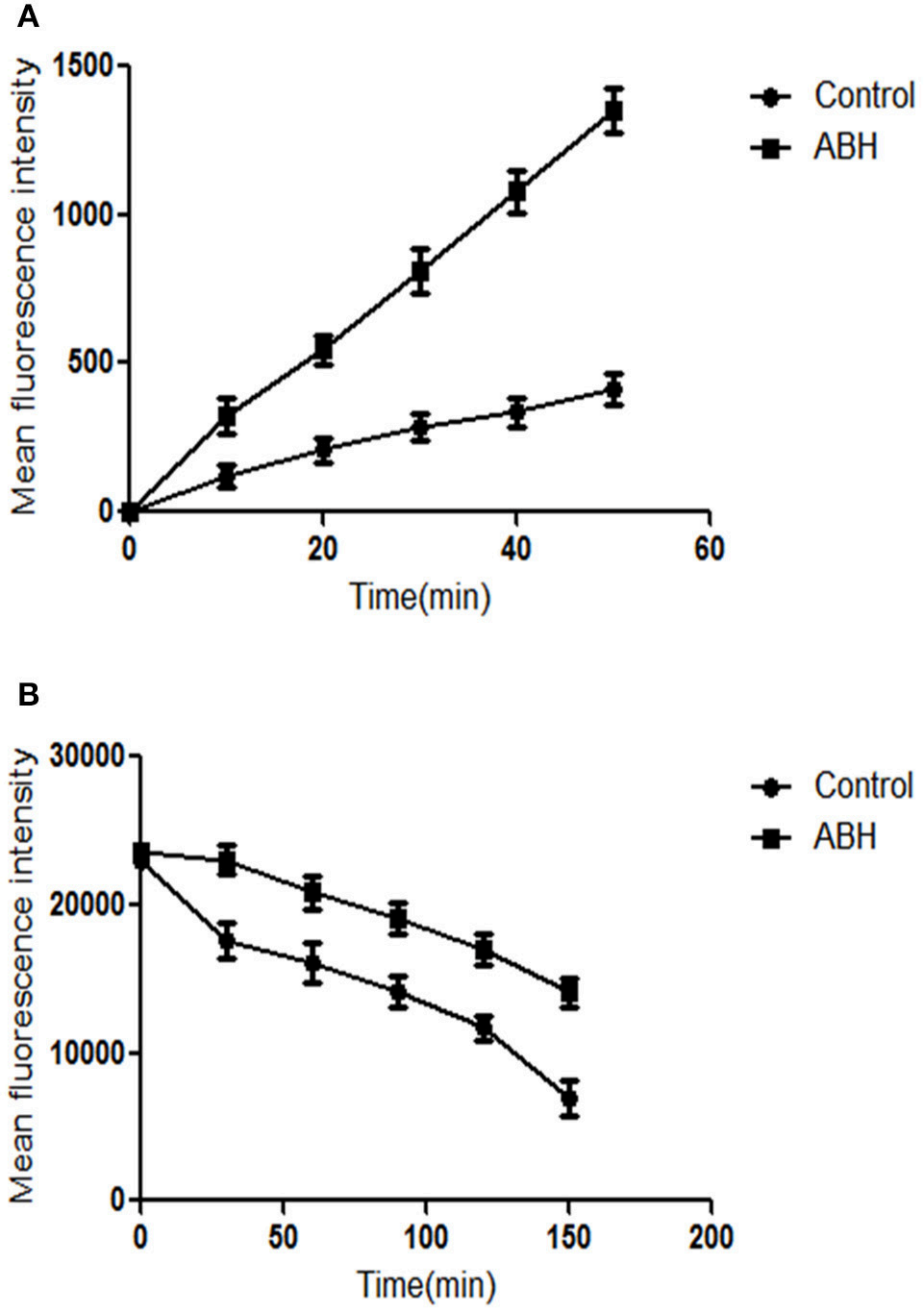
**The effect of ABH on the (A)** uptake and **(B)** efflux of Rh6G in resistant *C. albicans*. The uptake and efflux of Rh6G in the absence and presence of ABH (128 μg/mL) were determined by a flow cytometer. Ten thousand events were counted for each sample at specific time intervals. MFIs represent the intracellular Rh6G in *C. albicans*. GraphPad Prism 6 software was used to analyze the data. The experiments were performed three times on different days.

Both Rh6G and FLC are substrates of drug transporters in *C. albicans*. Rh6G was used as the alternate for FLC in the present study. We measured the uptake and efflux of Rh6G in resistant *C. albicans* in the absence and presence of ABH. Intracellular Rh6G uptake in the ABH-treated group increased over time, and that in the ABH group increased faster than that in the control group (Figure 4A). Cells treated with ABH pumped out lower concentrations of Rh6G than cells without drug treatment. This finding was evident by the sharp drop in the measured intracellular concentrations of Rh6G in the ABH-treated group (Figure 4B). Our results revealed that ABH can increase the uptake and decrease the efflux of rhodamine 6G.

## Discussion

In this study, we first examined the effects of ABH combined with FLC against planktonic *Candida* spp. cells with different susceptibility and biofilms of resistant *C. albicans* in different stages. Our results showed that the combination of FLC at a low concentration (2 μg/mL) with ABH at a concentration of 128 μg/mL exerts synergistic effects against resistant *C. albicans* only, with no synergism on susceptible *C. albicans* and non-*C. albicans*. ABH combined with FLC also showed synergistic antifungal effects against 4, 8, and 12 h biofilms of resistant *C. albicans*. These findings indicate that ABH shows a potent antifungal effect against the planktonic cells and biofilms of FLC-resistant *C. albicans in vitro* when combined with FLC.

Although mouse models are considered a standard model to investigate fungal pathogenesis, their disadvantages, such as cost, logistical challenges, and the fact that they are time-consuming and ethically delicate, have limited their application for *in vivo* experimentation (Amorim-Vaz et al., 2015). Therefore, a number of simple insect models have recently been developed for use with fungal pathogens. For example, *G. mellonella* has been increasingly used to study fungal virulence and the antifungal activity of drug combinations (Mylonakis et al., 2005; Vu and Gelli, 2010). In the present study, treatment of *G. mellonella* larvae infected by *C. albicans* with a combination of FLC and ABH exhibited greater efficacy than FLC or ABH monotherapy, which was in accordance with the synergistic antifungal effects of this combination *In vitro*. In addition, this study demonstrated the utility of the *G. mellonella* larva model as an inexpensive, rapid *in vivo* model to prescreen potential novel antifungal therapies prior to more extensive mammalian animal models. Although, the concentration of ABH required to induced the *in vivo* effects may be higher than the usual doses that ABH achieves in humans, high doses of ABH are widely used in clinics, and a large number of clinical trials have proven that treatment with high doses of ABH (more than 15 mg/kg or 1,000 mg/day) appears to improve the condition of patients with acute or mild respiratory distress syndrome and acute lung injury (Wu et al., 2014). Therefore, the ABH concentration used in this study still has the potential to be used clinically.

Azole-resistant isolates commonly display reduced intracellular accumulation of antifungal agents due to their over-expression of drug transport pumps (Prasad and Rawal, 2014). Two main classes of drug transport pumps are responsible for the development of antifungal resistance: ATP-binding cassette transporters and major facilitator superfamily transporters (Holmes et al., 2016). Considering the importance of major antifungal transport pumps, one focus of recent research has been to understand the relationship between this synergism and drug transport pumps. Functional activities of drug transport pumps have traditionally been assayed with Rh6G, a known fluorescent substrate of several drug transport pumps responsible for multidrug resistance in yeasts and a FLC alternate (Ahmad et al., 2013). We employed this Rh6G-based assay to determine whether the synergistic effects were correlated with the inhibition of drug transport pumps. ABH was found to significantly increase the uptake of Rh6G and decrease the efflux of Rh6G, indicating that ABH improves the susceptibility of resistant *C. albicans* to FLC by increasing the intracellular concentration of FLC. Inhibiting the effects on drug transport pumps may be one of the synergism mechanisms of ABH and FLC in combination. Other potent mechanisms need to be further investigated.

## Conclusion

We suggest that combinations of antifungal drugs with other, non-antifungal drugs, could be used as a useful approach for overcoming drug resistance in *C. albicans*. In this study, we found that combined FLC and ABH treatment results in synergistic effects against FLC-resistant *C. albicans in vitro* and *in vivo*. In addition, this combination also exhibited synergistic effects against resistant *C. albicans* biofilms in different stages. When looking for the mechanism of action of this drug combination, we could not rely on any known mechanism of ABH against *C. albicans*. Therefore, we first assayed the possibility that ABH acts on the most common FLC resistance mechanism in *C. albicans*, namely changes in drug uptake and efflux. Remarkably, we found that ABH can increase the uptake and decrease the efflux of a test compound, presumably by inhibiting drug transporters. This leads us to suggest that the synergy of ABH with FLC on drug-resistant *C. albicans* results from an increase in intracellular FLC concentration. Our results are thus the first to show that ABH, in combination with FLC, can be exploited to reverse the drug resistance of *C. albicans*. Therapeutic combination of ABH with FLC may constitute a new alternative in the treatment of infections by resistant *C. albicans*.

## Author contributions

XL, YZ, XH, CY, YY and SS performed the experiments. XL and SS designed the research. XL analyzed the data and wrote the paper. All authors approved the manuscript for publication.

## Funding

This work was supported by 2016GSF201187 from the Department of Science and Technology of Shandong Province of China. The funders had no role in the study design, data collection and analysis, decision to publish, or preparation of the manuscript.

### Conflict of interest statement

The authors declare that the research was conducted in the absence of any commercial or financial relationships that could be construed as a potential conflict of interest.

## Abbreviations

FLC: Fluconazole
ABH: Ambroxol hydrochloride
CA: *C. albicans*
YPD: Yeast–peptone–dextrose
*G. Mellonella*: *Galleria mellonella*
PAS: Periodic acid Schiff reagent
Rh6G: Rhodamine 6G
MFI: Mean fluorescent intensity.
