# Supplementary material for: Ambroxol Hydrochloride Combined with Fluconazole Reverses the Resistance of Candida albicans to Fluconazole
Source: Front Cell Infect Microbiol. 2017 Apr 7;7:124. doi: 10.3389/fcimb.2017.00124 (PMC5383865; doi:10.3389/fcimb.2017.00124)
Supplement: Supplementary file 1 [file Table1.DOCX]

S1 Table. Full checkerboard assay results for CA10 (A) and CA16 (B). Datum is the the mean of three independent experiments.

A

|  | | FLC(μg/mL) | | | | | | | | | | | |
| --- | --- | --- | --- | --- | --- | --- | --- | --- | --- | --- | --- | --- | --- |
|  |  | 0 | 0.125 | 0.25 | 0.5 | 1 | 2 | 4 | 8 | 16 | 32 | 64 | 空白 |
| ABH (μg/mL) | 256 | 60.41% | 0.97% | 0.75% | 0.89% | 0.56% | 0.48% | 0.57% | 0.22% | 0.85% | 0.47% | 0.22% | 0% |
|  | 128 | 94.11% | 78.00% | 64.88% | 29.90% | 27.96% | 14.44% | 11.87% | 9.51% | 7.75% | 4.44% | 0.35% | 0% |
|  | 64 | 95.70% | 78.60% | 67.99% | 61.49% | 58.34% | 43.15% | 42.35% | 41.89% | 40.77% | 39.56% | 37.48% | 0% |
|  | 32 | 96.34% | 80.52% | 69.75% | 65.01% | 62.60% | 60.54% | 57.33% | 59.17% | 56.02% | 54.42% | 50.43% | 0% |
|  | 16 | 96.84% | 80.95% | 70.28% | 63.89% | 63.47% | 63.24% | 57.67% | 59.54% | 56.59% | 54.81% | 51.22% | 0% |
|  | 8 | 96.66% | 81.73% | 73.36% | 64.69% | 62.99% | 63.78% | 60.37% | 60.60% | 58.42% | 57.08% | 53.77% | 0% |
|  | 4 | 99.05% | 82.25% | 75.09% | 66.57% | 64.15% | 63.94% | 60.27% | 61.46% | 59.18% | 55.96% | 55.41% | 0% |
|  | 0 | 100.00% | 82.42% | 76.84% | 70.09% | 66.91% | 65.34% | 62.17% | 61.75% | 60.50% | 59.03% | 57.26% | 0% |

B

|  | | FLC(μg/mL) | | | | | | | | | | | |
| --- | --- | --- | --- | --- | --- | --- | --- | --- | --- | --- | --- | --- | --- |
|  |  | 0 | 0.125 | 0.25 | 0.5 | 1 | 2 | 4 | 8 | 16 | 32 | 64 | 空白 |
| ABH (μg/mL) | 256 | 72.06% | 1.21% | 0.83% | 0.79% | 0.66% | 0.67% | 0.62% | 0.56% | 0.35% | 0.44% | 0.21% | 0% |
|  | 128 | 79.66% | 62.70% | 32.28% | 28.31% | 26.38% | 8.00% | 8.07% | 8.35% | 0.31% | 0.08% | 0.13% | 0% |
|  | 64 | 81.48% | 75.10% | 69.85% | 69.05% | 68.01% | 64.67% | 62.71% | 61.20% | 57.97% | 56.51% | 55.22% | 0% |
|  | 32 | 83.25% | 78.59% | 76.96% | 71.40% | 69.10% | 68.24% | 65.07% | 62.29% | 60.51% | 58.45% | 60.94% | 0% |
|  | 16 | 96.32% | 79.32% | 77.85% | 73.55% | 69.45% | 69.01% | 66.13% | 65.27% | 64.80% | 62.87% | 61.83% | 0% |
|  | 8 | 97.40% | 80.03% | 81.11% | 74.66% | 70.55% | 70.91% | 66.87% | 66.85% | 65.94% | 64.37% | 62.17% | 0% |
|  | 4 | 97.00% | 80.29% | 82.23% | 75.49% | 72.51% | 71.51% | 68.53% | 69.93% | 67.37% | 65.60% | 64.78% | 0% |
|  | 0 | 100.00% | 83.54% | 84.01% | 78.58% | 72.56% | 70.52% | 70.24% | 69.61% | 69.86% | 67.85% | 65.65% | 0% |
